# Identification of Genome-Wide Variations among Three Elite Restorer Lines for Hybrid-Rice

**DOI:** 10.1371/journal.pone.0030952

**Published:** 2012-02-23

**Authors:** Shuangcheng Li, Shiquan Wang, Qiming Deng, Aiping Zheng, Jun Zhu, Huainian Liu, Lingxia Wang, Fengyan Gao, Ting Zou, Bin Huang, Xuemei Cao, Lizhi Xu, Chuang Yu, Peng Ai, Ping Li

**Affiliations:** 1 Rice Research Institute, Sichuan Agricultural University, Wenjiang, Sichuan, China; 2 Key Laboratory of Crop Genetic Resources and Improvement, Ministry of Education, Sichuan Agricultural University, Ya'an, Sichuan, China; 3 State Key Laboratory of Hybrid Rice, Sichuan Agricultural University, Chengdu, China; United States Department of Agriculture, United States of America

## Abstract

Rice restorer lines play an important role in three-line hybrid rice production. Previous research based on molecular tagging has suggested that the restorer lines used widely today have narrow genetic backgrounds. However, patterns of genetic variation at a genome-wide scale in these restorer lines remain largely unknown. The present study performed re-sequencing and genome-wide variation analysis of three important representative restorer lines, namely, IR24, MH63, and SH527, using the Solexa sequencing technology. With the genomic sequence of the Indica cultivar 9311 as the reference, the following genetic features were identified: 267,383 single-nucleotide polymorphisms (SNPs), 52,847 insertion/deletion polymorphisms (InDels), and 3,286 structural variations (SVs) in the genome of IR24; 288,764 SNPs, 59,658 InDels, and 3,226 SVs in MH63; and 259,862 SNPs, 55,500 InDels, and 3,127 SVs in SH527. Variations between samples were also determined by comparative analysis of authentic collections of SNPs, InDels, and SVs, and were functionally annotated. Furthermore, variations in several important genes were also surveyed by alignment analysis in these lines. Our results suggest that genetic variations among these lines, although far lower than those reported in the landrace population, are greater than expected, indicating a complicated genetic basis for the phenotypic diversity of the restorer lines. Identification of genome-wide variation and pattern analysis among the restorer lines will facilitate future genetic studies and the molecular improvement of hybrid rice.

## Introduction

As the main staple food for more than half of the world's population, rice (*Oryza sativa* L.) is one of the most important food crops. In 1973, the field production of Indica hybrid rice succeeded when Chinese rice breeders completed the three-line breeding system [1]. A land area of approximately 130,000 hm^2^ was soon developed for hybrid rice cultivation, greatly increasing rice yield in China. In the three-line breeding system, the cytoplasmic male sterility (CMS) line is crossed with the restorer line to produce the F_1_ hybrid rice, and with the maintainer line for self-reproduction. The restorer line is widely considered as being key to further improve the resistance, yield, quality, and heterosis of hybrid rice [1], [2]. IR24, an elite rice variety introduced in China by the International Rice Research Institute, was the most common restorer line used during the 1970s until the early 1980s. MH63, which was developed from a cross between IR30 and Gui630 [1], is thus far the most widely used restorer line in China. Its popularity can be attributed to the fact of being a co-parent of ShanYou63, the largest hybrid rice acreage that has created substantial economic and social benefits. SH527 is a heavy-panicle restorer line bred in the 1990s [3]. More than 40 new elite hybrid rice varieties have been bred using SH527 as the male parent, among which 5 were chosen for super hybrid rice development. At present, many hybrid rice varieties generated from SH527 are widely grown in China. IR24, MH63, and SH527 thus represent the first-, second-, and third-generation restorer lines, respectively, of the three-line breeding system. Although they are all significant backbone parents at different stages of hybrid rice development, their field performances and combining abilities differ considerably. Further research on the genetic diversity of these lines, which might be related to their varying performances, can improve our understanding of restorer lines and promote improved restorer line selection and super hybrid rice breeding.

The genomic sequences of the Japonica cultivar Nipponbare [4] and the Indica cultivar 9311 [5] were recently released. The availability of high-throughput sequencing technology not only increases sequencing throughput but also allows for simultaneous sequencing of a large number of samples [6], [7] in addition to decreasing time and cost. These merits open the door to high-throughput re-sequencing and genotyping of various rice strains. A genetic map with a resolution of recombination breakpoints within an average of 40 kb were previously constructed for ∼150 rice recombinant inbred lines by utilizing whole-genome re-sequencing data generated using the Illumina Genome Analyzer [8]. Six elite maize inbred lines, including the parents of the most productive commercial hybrid in China, were recently re-sequenced and more than 1,000,000 SNPs, 30,000 indel polymorphisms and 101 low-sequence-diversity chromosomal intervals were uncovered in the maize genome [9]. Huang et al. [10] identified approximately 3.6 million single-nucleotide polymorphisms (SNPs) by sequencing 517 rice landraces and constructed a high-density haplotype map of the rice genome. Moreover, they pioneered genome-wide association studies for 14 agronomic traits of the *O. sativa indica* subspecies. Molecular marker screening has suggested narrow genetic backgrounds for rice restorer lines [11], [12], which play a vital role in hybrid rice production. However, the current lack of information on genetic variation over the entire genome has limited further research into this topic. In the present study, we conducted re-sequencing and genome-wide variation analysis of IR24, MH63, and SH527 using the Solexa sequencing technology. Identification of genome-wide single-nucleotide polymorphisms (SNPs), insertion/deletion polymorphisms (InDels), and structural variations (SVs) as well as pattern analysis among these lines has the potential to provide valuable resources for future genetic studies and the molecular improvement of hybrid rice.

## Results

### Field performances of the restorer lines and their hybrid descendants

**Figure 1:**
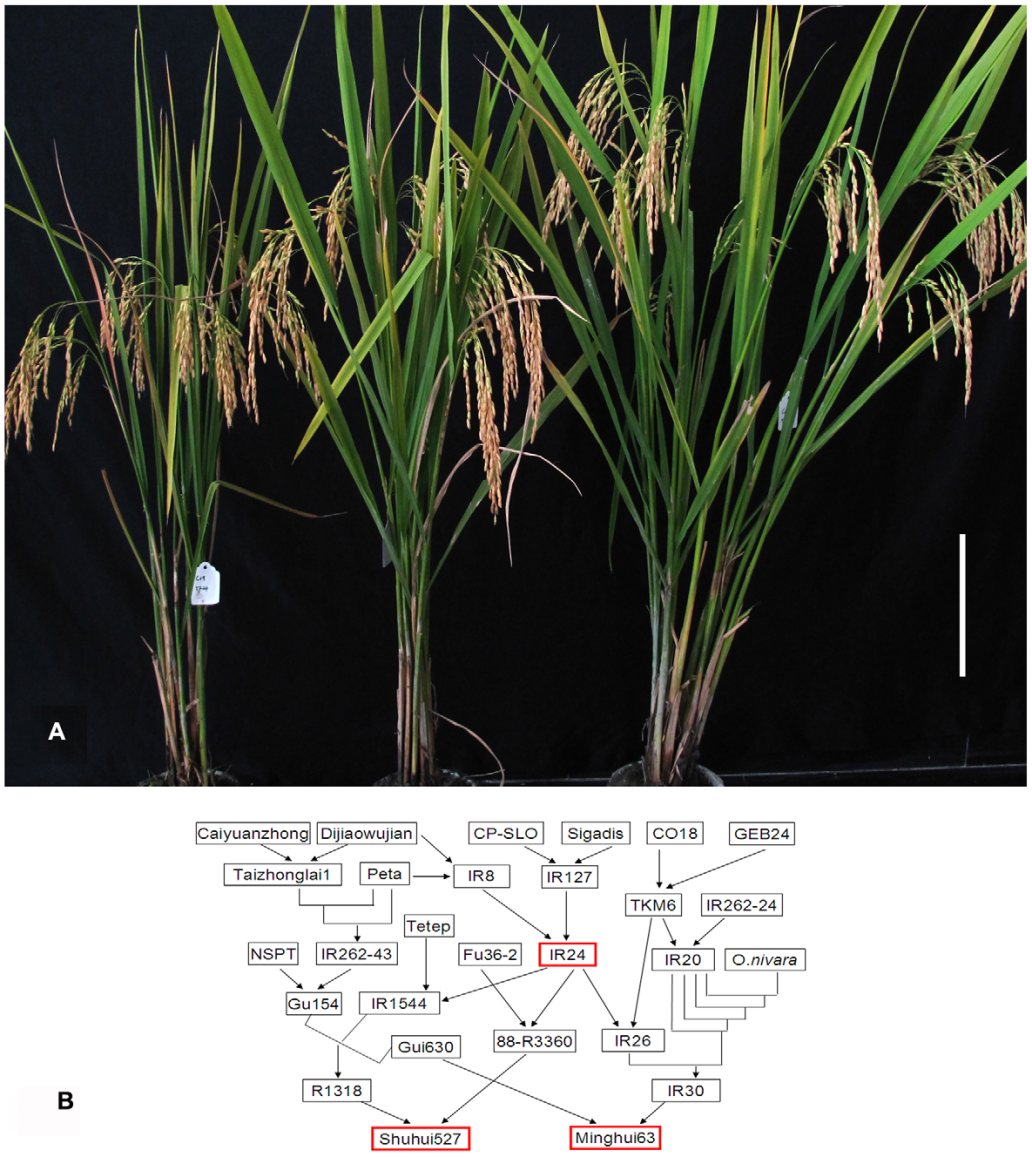
Plant phenotypes of the three core restorer lines and their cross genealogies. a, Plant phenotypes of the three core restorer lines; shown from left to right are IR24, MH63, and SH527. b, Cross genealogies of the three core restorer lines showing that MH63 and SH527 were indirectly generated from IR24. A new rice line (at the head of an arrow) was bred by crossing of two or more parents (at the tail of an arrow) and by several turns of subsequent selfing and selection. A straight line in the figure indicates a backcross.

IR24, MH63, and SH527 (Fig. 1A) are considered hybrid rice core restorer lines because of the large number of elite commercial hybrid rice cultivars and useful restorer lines bred and generated from them. Based on their cross genealogies, MH63 and SH527 were both indirectly generated from IR24 (Fig. 1B), indicating that these three lines originate from the same restoring genes. We examined the field performances of these lines by selfing (Table 1). Performances of the hybrid rice made by crossing these three lines with six other widely used CMS lines, namely, G3A, Zhongjiu A, II-32A, G46A, 92A, and Chuangu A, were also examined (Table 1). No obvious differences were found in the yield components of MH63 and IR24 except for plant height, while the hybrid rice of MH63 was significantly different from that of IR24 in growth period, plant height, panicles per plant and seed setting rate. Between SH527 and IR24, significant differences were detected in plant height, panicles per plant and 1000-grains weight. Significant differences between their hybrid rice were also detected in growth period, plant height, seed setting rate and 1000-grains weight. In general, from the breeding stage of IR24, MH63 to SH527, combinations of these changes lead to an apparent yield increase for hybrid rice, although no obvious yield differences were found in the restorer lines themselves. Since the yield increase was evaluated on the average performance of hybrid rice generated from these three restorer lines with several common CMS lines, the yield increase of hybrid rice reflect an obvious genetic improvement of the restorer lines, possibly by improving the combining ability of the restorer lines.

**Table 1:** Field performance of 3 restorer lines.

line	Growth period (d)		Plant height (cm)		Panicles per plant		Grains per panicle		Seed setting rate (%)		Weight per 1000-grains (g)		Yield (Kg/Mu)	
	inbred	hybrid	inbred	hybrid	inbred	hybrid	inbred	hybrid	inbred	hybrid	inbred	hybrid	inbred	hybrid
**IR24**	108.50±0.71^a^	111.25±1.66^a^	89.00±2.83^b^	106.08±3.94^b^	14.03±0.53^a^	11.84±0.97^b^	148.00±7.07^a^	207.08±24.49^a^	87.50±0.71^ab^	78.17±7.46^b^	27.65±0.21^b^	26.63±1.39^b^	375.00±44.41^a^	442.26±39.95^c^
**MH63**	111.50±0.71^a^	110.00±1.81^b^	100.00±4.24^a^	117.92±5.28^a^	15.68±0.53^a^	14.25±2.34^a^	128.95±8.69^a^	197.58±26.02^a^	81.50±2.12^b^	82.42±4.80^a^	28.50±0.70^b^	27.03±0.89^b^	354.00±14.28^a^	467.57±50.08^b^
**SH527**	109.00±1.41^a^	110.50±1.57^b^	109.00±0.00^a^	119.00±4.79^a^	10.58±0.95^b^	11.80±1.41^b^	151.95±8.84^a^	205.59±17.60^a^	93.50±4.95^a^	83.42±3.75^a^	32.80±0.71^a^	28.81±1.29^a^	367.05±59.46^a^	498.78±21.68^a^

Note that the inbred lines and hybrids were analyzed by One-way ANOVA and Two-way ANOVA method, respectively, and the superscript letters a,b and c indicate significant difference detected by LSD's test at P<0.05.

### Genome sequencing and variation identification

The genotypes of IR24, MH63, and SH527 were determined with approximately 10-fold coverage by genome sequencing using the Solexa sequencing technology. According to the protocol, three DNA libraries were constructed and 12.48 G bases were generated (raw sequence data obtained have been deposited in the NCBI Short Read Archive with accession number SRP006823). The alignment of reads was used to build consensus genome sequences for each rice accession. Furthermore, approximately 10.78 G high-quality raw databases were aligned with the reference sequence of cultivar 9311 using SOAPaligner [13] (http://soap.genomics.org.cn/). In total, an effective depth of 30× coverage was achieved, with an average of 10× for each restorer line (Table 2). The resulting consensus sequence of each rice accession covered approximately 84.8% of the reference genome (84%–85.99%), indicating a close relationship between the samples and cultivar 9311.

**Table 2:** Summary of original sequencing data.

Sample	Insert size	Bases (G)	Mapped Bases (G)	Depth	Coverage(%)	Mismatch Rate(%)
**IR24**	474	4.87	4.28	11.92	85.99	0.60
**MH63**	473	3.79	3.22	8.97	84.0	0.75
**SH527**	468	3.82	3.28	9.12	84.43	0.69

**Figure 2:**
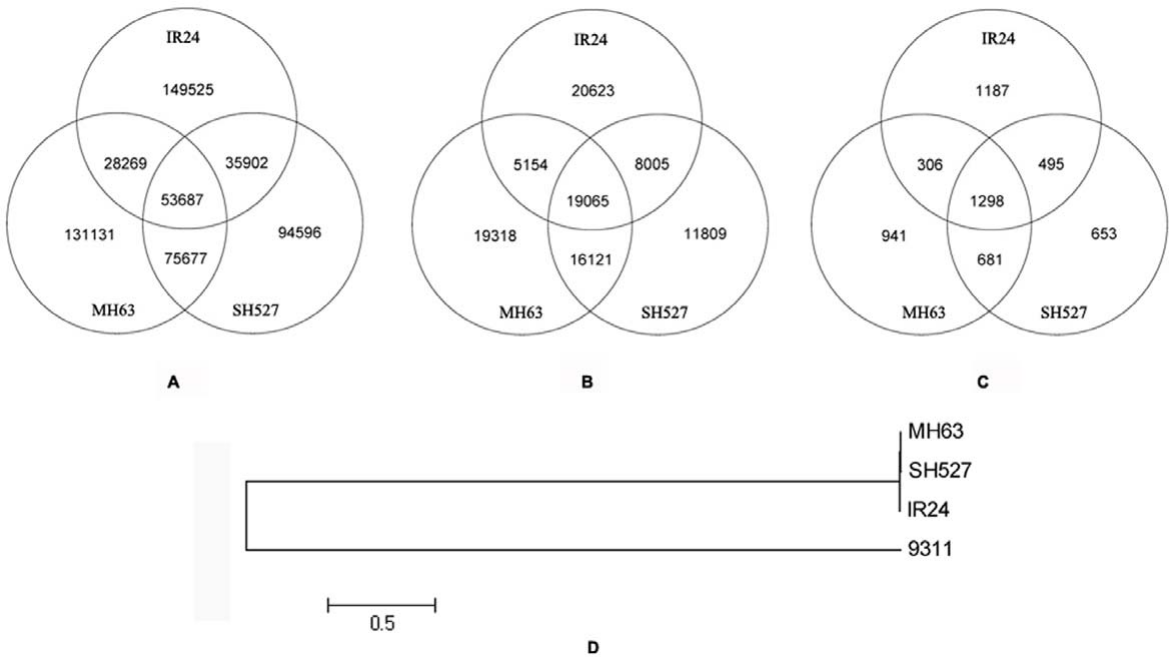
Shared variation clusters among IR24, MH63, and SH527 and phylogenetic tree analysis. a–c, Synteny analysis results for (a) SNPs, (b) InDels, and (c) SVs. d, Phylogenetic tree constructed by authentic collections of SNPs.

SNPs, InDels, and SVs were then examined with SOAPsnp11 and SOAPsv using a conservative quality filter pipeline [14], yielding 267,383 SNPs from the genome of IR24, 288,764 SNPs from that of MH63, and 259,862 SNPs from that of SH527 (Table 3, http://rice.sicau.edu.cn/re-sequencing/variation/9311.rar). These outcomes resulted in a non-redundant collection of 568,787 SNPs after excluding the shared SNPs of each sample by synteny analysis (Fig. 2A–2C). In total, 100,095 InDels ranging from 1 to 5 bp in length and 5,561 SVs across the whole genome were identified. Because of inherent relationship between the samples, the overall genome diversity among these re-sequenced elite restorer lines was much lower than that reported for a more diverse population [10], which is also in accordance with the close relationship among the three lines revealed by genealogy analysis. A phylogenetic tree [15] was constructed using several authentic collections of SNPs. An extremely closed genetic relationship was observed between sequencing samples, and a relatively distant relationship was observed between samples and the reference (Fig. 2D), which is consistent with a previously reported result of low genome diversity among rice restorer lines [11], [12].

**Table 3:** Variations detected for each sample.

Chromosome	SNPs			InDels			SVs		
	IR24	MH63	SH527	IR24	MH63	SH527	IR24	MH63	SH527
**Chr01**	36,134	34,498	33,949	8,026	7,755	7,779	528	488	491
**Chr02**	25,139	36,400	29,835	5,258	8,064	6,868	215	274	246
**Chr03**	19,810	30,599	27,263	4,249	7,097	6,519	322	363	352
**Chr04**	19,042	26,016	22,324	3,413	4,986	4,259	273	276	284
**Chr05**	32,928	21,990	21,396	6,212	4,726	4,638	283	241	232
**Chr06**	16,015	24,585	25,190	3,187	4,880	5,247	233	261	262
**Chr07**	12,093	13,607	10,325	2,061	2,388	1,807	98	108	88
**Chr08**	29,097	27,334	26,804	5,564	5,501	5,491	423	382	390
**Chr09**	17,905	13,685	13,451	3,657	2,723	2,891	153	115	120
**Chr10**	21,873	16,421	14,736	4,281	3,315	3,105	269	201	189
**Chr11**	19,377	19,470	16,873	3,472	3,625	3,329	297	267	252
**Chr12**	17,970	24,159	17,716	3,467	4,598	3,567	192	250	221
**Total**	**267,383**	**288,764**	**259,862**	**52,847**	**59,658**	**55,500**	**3,286**	**3,226**	**3,127**

**Figure 3:**
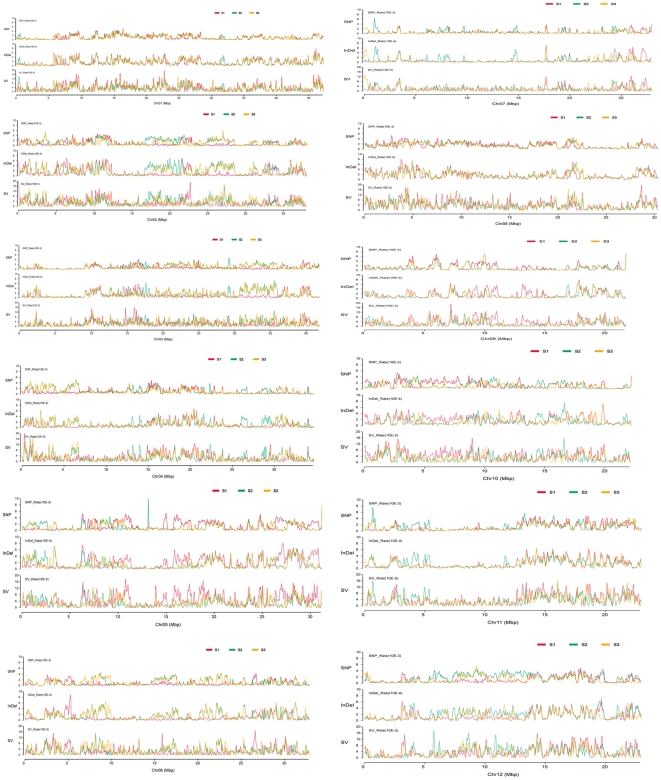
Comparative distributions of variation frequency on 12 chromosomes. s1, IR24; S2, MH63; S3,SH527.

**Figure 4:**
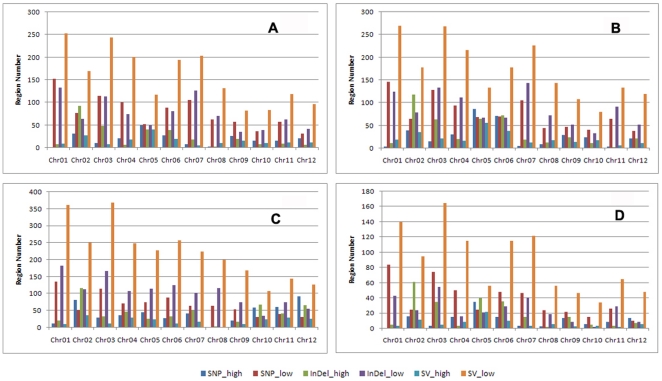
High and low regions of variation between samples. a, IR24 vs. MH63. b, IR24 vs. SH527. c, MH63 vs. SH527. d, IR24 vs. MH63 vs. SH527.

The frequencies of SNPs, InDels, and SVs for each sample were plotted at a 100 kb sliding window with a step size of 50 kb along each chromosome. SNP/InDel/SV frequency was defined as the corresponding number of SNPs/InDels/SVs divided by the number of nucleotides within the 100 kb interval, excluding the uncovered nucleotides. Each sample was compared with the corresponding intervals to identify regions that showed non-random variation frequencies. In total, 227/936 SNP high/low regions, 298/889 InDel high/low regions, and 188/1899 SV high/low regions were identified between IR24 and MH63; 339/914 SNP high/low regions, 440/1,030 InDel high/low regions, and 267/2,052 SV high/low regions were identified between IR24 and SH527; and 507/825 SNP high/low regions, 523/1,266 InDel high/low regions, and 235/2,684 SV high/low regions were identified between MH63 and SH527. Out of these, 135/450 SNP high/low regions, 229/297 InDel high/low regions, and 87/1,058 SV high/low regions were found to be identical among the three restorer lines (Figs. 3 and 4).

### Variations between samples

As differences between the samples (i.e., not between the samples and the reference) may reflect the genetic improvement of the recent restorer lines (such as SH527 and MH63) from older lines (such as IR24), an analysis of the variations and their distributions among the samples was performed. Synteny analysis of variations revealed 81,956 shared SNPs, 2,799 different SNPs, 24,053 shared InDels, and 860 different InDels between IR24 and MH63; 89,589 shared SNPs, 3,998 different SNPs, 26,936 shared InDels, and 634 different InDels between IR24 and SH527; and 129,364 shared SNPs, 2,927 different SNPs, 35,066 shared InDels, and 613 different InDels between MH63 and SH527. The distributions of these variations on each chromosome are showed in Table 4. Furthermore, only 10 different SNPs and 12 different InDels (allelic pleomorphic loci with different nucleotides in each line) were identified by the variation consensus comparative analysis of the three sequenced lines, although large numbers of shared SNPs and InDels were found (Table 5).

**Table 4:** Variations detected between each sample.

Chromosome	SNPs						InDels					
	IR24 vs MH63		MH63 vs SH527		IR24 vs SH527		IR24 vs MH63		MH63 vs SH527		IR24 vs SH527	
	Shared	Different	Shared	Different	Shared	Different	Shared	Different	Shared	Different	Shared	Different
**Chr01**	16776	364	17425	437	20167	191	5107	86	5380	70	5761	26
**Chr02**	6207	331	5548	470	13209	586	1833	162	1717	97	3747	170
**Chr03**	6268	216	6708	275	15437	333	2191	66	2410	51	4699	54
**Chr04**	5813	257	7138	618	11451	275	1597	49	2184	19	2732	41
**Chr05**	7075	239	7400	208	10666	121	2056	73	2058	88	3045	28
**Chr06**	5042	211	4631	229	13302	150	1663	17	1518	22	3541	23
**Chr07**	3697	92	3412	181	3964	169	1063	22	1017	15	979	17
**Chr08**	10698	271	10938	335	16565	109	2745	132	2847	117	4227	14
**Chr09**	3961	164	5089	398	6062	261	1218	56	1693	49	1655	32
**Chr10**	4809	234	5513	275	5085	152	1514	57	1808	50	1381	56
**Chr11**	5407	194	9257	150	6440	163	1430	71	2406	22	1594	58
**Chr12**	6203	226	6530	422	7016	417	1636	69	1898	34	1705	94
**Total**	**81956**	**2799**	**89589**	**3998**	**129364**	**2927**	**24053**	**860**	**26936**	**634**	**35066**	**613**

**Table 5:** Three sequenced lines shared/different variations.

Chromosome	SNPs		InDels	
	Shared	Different	Shared	Different
**Chr01**	12245	0	4336	0
**Chr02**	3255	2	1197	2
**Chr03**	4313	1	1841	2
**Chr04**	4082	2	1331	1
**Chr05**	4645	0	1592	0
**Chr06**	3141	1	1275	0
**Chr07**	2112	0	750	1
**Chr08**	7665	1	2339	0
**Chr09**	2493	0	979	0
**Chr10**	2460	0	965	5
**Chr11**	3772	2	1216	0
**Chr12**	3504	1	1121	1
**Total**	**53687**	**10**	**18942**	**12**

The SNPs in coding regions were analyzed to gain further insights into the potential functional effects of the detected SNPs (Table 6). Between IR24 and MH63, 13,160 shared SNPs, of which 2,290 were synonymous coding sequences (Syn CDS) and 2,902 were non-synonymous coding sequences (Non-syn CDS), and 291 different SNPs, of which 54 were Syn CDS and 99 were Non-syn CDS, were found. Between IR24 and SH527, 14,473 shared SNPs (2,522 Syn CDS and 3,366 Non-syn CDS) and 594 different SNPs (94 Syn CDS and 138 Non-syn CDS) were found in coding regions. Moreover, 22,096 shared SNPs (3,517 Syn CDS and 4,738 Non-syn CDS) and 417 different SNPs (76 Syn CDS and 97 Non-syn CDS) were found between MH63 and SH527. In total, 666, 705 and 735 shared CDS-located InDels were found between IR24 and MH63, IR24 and SH527, and MH63 and SH527, respectively (Table 7). Different CDS-located InDels were not detected.

**Table 6:** Syn_CDS and Non_syn_CDS SNPs variations between samples.

chromosome	IR24 vs MH63				IR24 vs SH527				MH63 vsSH527				IR24 vs MH63 vsSH527	
	Shared		Different		Shared		Different		Shared		Different		Shared	
	Syn_CDSs	Non_syn_CDSs	Syn_CDSs	Non_syn_CDSs	Syn_CDSs	Non_syn_CDSs	Syn_CDSs	Non_syn_CDSs	Syn_CDSs	Non_syn_CDSs	Syn_CDSs	Non_syn_CDSs	Syn_CDSs	Non_syn_CDSs
**Chr01**	454	559	5	11	475	592	15	8	561	699	1	4	343	419
**Chr02**	237	223	9	12	229	206	13	8	504	560	22	18	141	127
**Chr03**	144	221	3	6	172	246	3	10	327	513	5	7	106	170
**Chr04**	119	182	6	16	163	221	17	25	286	421	6	16	88	113
**Chr05**	168	220	4	10	206	251	0	16	250	322	4	5	127	154
**Chr06**	192	229	4	3	151	225	3	5	383	486	1	5	118	161
**Chr07**	118	158	0	3	94	139	4	7	97	161	4	5	61	92
**Chr08**	275	355	7	8	279	362	8	7	404	599	2	1	190	247
**Chr09**	99	149	1	4	143	195	12	23	150	212	4	12	71	104
**Chr10**	118	173	6	14	135	228	8	14	127	202	6	5	62	112
**Chr11**	188	243	1	6	291	419	0	4	221	290	5	8	138	168
**Chr12**	178	250	8	6	184	282	11	11	207	273	16	11	111	158
Total	2290	2962	54	99	2522	3366	94	138	3517	4738	76	97	1556	2025

**Table 7:** None-CDS and CDS located InDels variations between samples.

chromosome	IR24 vs MH63				IR24 vs SH527				MH63 vsSH527				IR24 vs MH63 vsSH527	
	Shared		Different		Shared		Different		Shared		Different		Shared	
	NONE-CDS	CDS	NONE-CDS	CDS	NONE-CDS	CDS	NONE-CDS	CDS	NONE-CDS	CDS	NONE-CDS	CDS	NONE-CDS	CDS
**Chr01**	1159	123	20	1	1212	128	15	1	1288	129	9	1	1033	108
**Chr02**	433	61	28	0	360	58	18	0	859	73	27	0	278	51
**Chr03**	524	69	14	0	550	71	8	0	1047	83	13	0	451	61
**Chr04**	383	63	13	0	499	68	3	0	615	76	12	0	324	55
**Chr05**	382	52	14	0	404	52	18	0	608	57	2	0	324	42
**Chr06**	398	48	3	0	381	43	2	0	801	54	5	0	329	37
**Chr07**	243	42	5	0	233	43	6	0	233	37	2	0	192	34
**Chr08**	548	56	25	0	554	62	21	0	791	77	3	0	464	49
**Chr09**	272	34	15	0	383	43	11	0	364	35	9	0	228	29
**Chr10**	303	41	11	0	371	41	6	0	281	41	16	0	219	34
**Chr11**	286	36	11	0	462	54	3	0	288	38	10	0	242	30
**Chr12**	311	41	9	0	358	42	2	0	367	35	19	0	223	29
Total	5242	666	168	1	5767	705	113	1	7542	735	127	1	4307	559

Three hundred thirty-one large-effect SNPs that were expected to affect the integrity of encoded proteins were also identified. These included changes introduced by premature termination codons (premature termination; 238 SNPs), elimination of translation initiation sites (ATG change; 11 SNPs), and replacement of nonsense with sense codons (stop change; 82 SNPs). Of these large-effect SNPs, only 10 SNPs (2 ATG changes, 5 premature terminations, and 3 stop changes) were observed from the different SNPs; the rest were from the shared SNPs (Table 8).

**Table 8:** large-effect SNPs between samples.

Chromosome	IR24 vs MH63			IR24 vs SH527			MH63 vsSH527			IR24 vs MH63 vs SH527		
	ATG change	Premature STOP	STOP change	ATG change	Premature STOP	STOP change	ATG change	Premature STOP	STOP change	ATG change	Premature STOP	STOP change
**Chr01**	0	8	3	0	8	4	0	12	4	0	7	2
**Chr02**	4	3	3	1	2	1	0	4	3	0	0	1
**Chr03**	1	5	2	1	7	2	0	13	4	0	4	2
**Chr04**	0	4	3	1	6	3	0	8	3	0	3	2
**Chr05**	0	7	1	0	9	1	0	5	4	0	5	1
**Chr06**	0	2	0	0	3	0	0	5	0	0	1	0
**Chr07**	0	1	0	0	0	0	0	2	0	0	0	0
**Chr08**	0	6	2	0	9	2	0	14	2	0	5	2
**Chr09**	0	5	1	0	7	1	0	6	2	0	3	1
**Chr10**	0	6	3	0	7	1	1	5	3	0	3	1
**Chr11**	0	2	2	0	6	3	0	2	2	0	0	1
**Chr12**	0	1	1	0	5	0	0	6	0	0	1	0
Total	5	50	21	3	69	18	1	82	27	0	32	13

**Figure 5:**
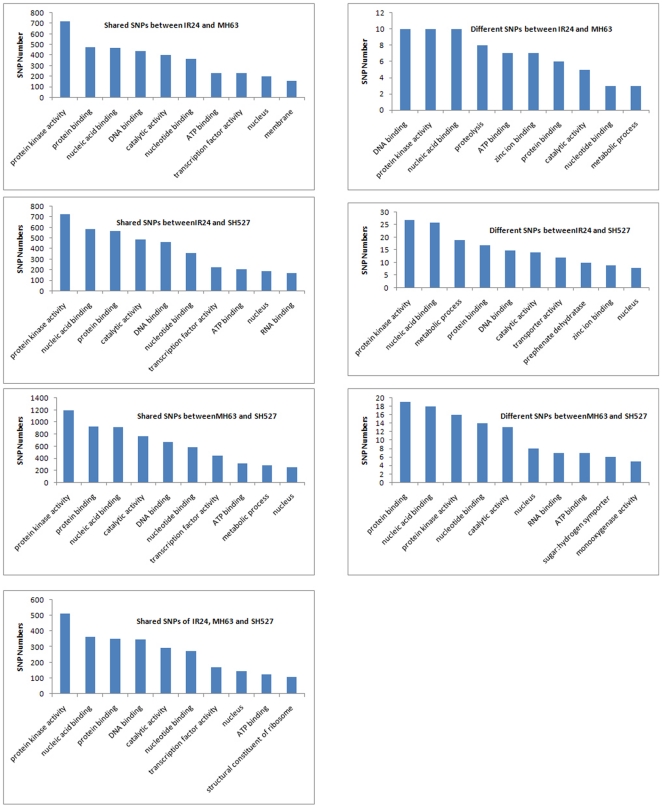
Top 10 GOs of SNPs detected between samples.

**Figure 6:**
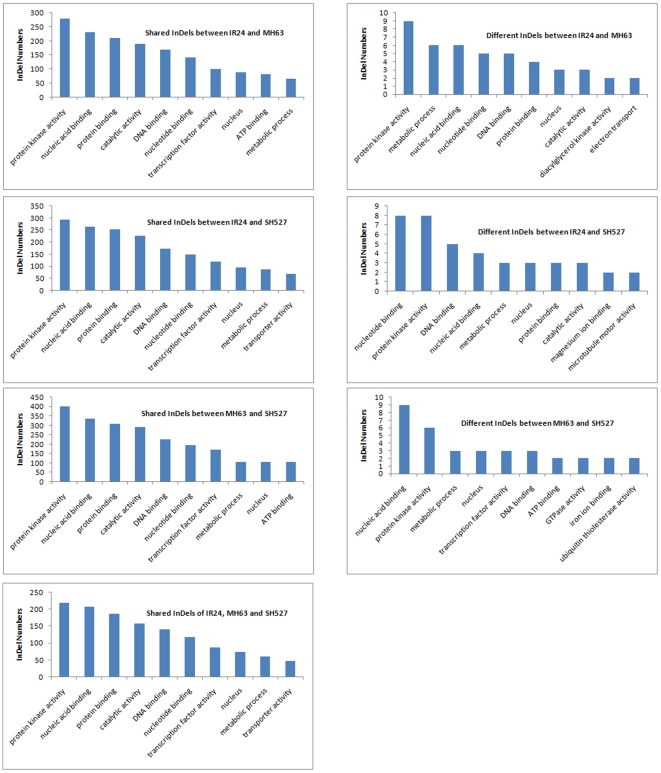
Top 10 GOs of InDels detected between samples.

GO and PFAM analyses were further carried out for the shared and different SNPs (InDels) in genes between samples to explore gene functions. In both the shared and different SNPs (InDels), the top GOs were protein kinase activity, nucleic acid binding, protein binding, DNA binding, and catalytic activity (Fig. 5 and 6). Genes coding for leucine-rich repeats and NB-ARC domains were found to have a significantly higher ratio of nonsynonymous-to-synonymous SNPs than average. As these domains are common in proteins that mediate disease resistance in plants, our finding is consistent with these proteins being particularly diverse due to pathogen pressure.

### Variation analysis on important rice genes

Several important rice genes related to yield, quality, resistance, and development processes were subjected to molecular cloning and functional analysis. Natural variations among the genes, which might explain the phenotypic differences of the sequenced sample, were then evaluated. A large number of SNPs (Table 9) were detected both in the DNA sequence and in the coding regions of genes related to disease/insect resistance, such as *Pib* [16], *Xa1* [17], *Pi9* [18], *Xa21* [19], *Xa26* [20] and *Bph14* [21]. Although found to have many SNPs, genes related to rice developmental processes, yield, and quality, such as *ALK* [22], *qSW5* [23], *GS3* [24], *Gn1a* [25], *HTD2* [26], *GW2* [27] and *EUI1* [28], had rare or no variations in the coding regions, which might explain the functional conservation. In addition, only a few InDels (or none in some cases) were found in the coding regions (Table 10), suggesting that SNPs, not InDels, effectively contribute to functional variation of the genes. When compared to the 9311 sequence, a number of SNPs were found both in the DNA sequence (∼60) and in the coding regions (∼40) of *Rf1a* [29], a possible allelic gene for *Rf4* [30], which is the major restoring gene of the WA-CMS line, while the sequence difference in this gene between the sequencing samples was limited. These variations may account for the differences between the sequenced samples (restorer lines) and the reference cultivar 9311(non-restorer lines) in terms of their restoring ability.

**Table 9:** Cloned rice gene SNP detect in IR24, MH63 and SH527.

Gene	IR24		MH63		SH527	
	DNA	mRNA	DNA	mRNA	DNA	mRNA
*ALK*	10	0	4	0	3	0
*Bph14*	16	8	5	3	7	6
*DWARF10*	3	12	1	7	0	0
*DWARF27*	15	5	10	5	13	5
*DEP1*	10	1	7	0	6	0
*EUI1*	5	0	3	0	9	0
*OsPPDKB*	24	3	38	1	34	2
*GIF1*	13	2	8	1	5	1
*Gn1a*	11	0	9	0	8	0
*GS3*	23	0	19	0	17	0
*GW2*	11	1	12	1	10	0
*HTD2*	8	0	6	0	9	0
*LAX*	1	1	0	0	0	0
*MOC1*	3	1	11	1	13	2
*OsGS1*	3	0	3	0	3	0
*OsMPK6*	6	0	4	0	4	0
*OsGT1*	18	0	13	0	16	0
*OsTB1*	0	0	1	1	1	1
*Pi21*	1	0	0	0	1	0
*Pi37*	7	0	2	2	7	5
*Pi9*	21	0	21	20	19	19
*Pib*	39	0	53	35	41	22
*Pi-d2*	14	0	14	0	17	0
*Pik-h*	7	7	1	1	1	1
*Pi-ta*	9	1	5	1	5	1
*qSW5*	47	0	27	0	41	0
*Rf1a*	63	40	60	36	65	37
*Rf1b*	2	2	5	4	2	1
*rTGA2.1*	6	2	8	3	7	3
*SaF*	5	2	4	0	5	2
*SaM*	9	0	5	0	6	0
*sd1*	1	0	0	0	1	0
*OsSSIIIa*	25	0	22	5	23	3
*Xa13*	7	0	3	0	6	0
*Xa1*	18	12	32	25	33	20
*Xa21*	11	10	29	29	10	11
*Xa26*	20	0	14	16	10	8
*Xa5*	28	0	20	0	27	0

**Table 10:** Cloned rice gene InDel detect in IR24, MH63 and SH527.

Gene	IR24		MH63		SH527	
	DNA	mRNA	DNA	mRNA	DNA	mRNA
*ALK*	2	0	0	0	0	0
*Bph14*	0	0	0	0	0	0
*DWARF10*	0	0	0	0	0	0
*DWARF27*	5	0	2	0	5	0
*DEP1*	0	0	1	0	1	0
*EUI1*	1	0	1	0	3	0
*OsPPDKB*	7	0	14	0	15	0
*GIF1*	2	0	3	0	1	0
*Gn1a*	3	0	2	0	1	0
*GS3*	8	0	9	0	9	0
*GW2*	3	0	3	0	4	0
*HDT2*	3	0	5	0	4	0
*LAX*	0	0	0	0	0	0
*MOC1*	1	0	0	0	0	0
*OsGS1*	2	0	0	0	2	0
*OsMPK6*	2	0	2	0	1	0
*OsGT1*	0	0	1	0	0	0
*OsTB1*	0	0	0	0	0	0
*Pi21*	0	0	0	0	0	0
*Pi37*	0	0	0	0	0	0
*Pi9*	0	0	0	0	0	0
*Pib*	0	0	2	0	3	0
*Pi-d2*	0	0	0	0	0	0
*Pik-h*	0	0	0	0	1	0
*Pi-ta*	0	0	0	0	1	0
*qSW5*	10	0	9	0	8	0
*Rf1a*	1	0	1	0	1	0
*Rf1b*	0	0	0	0	0	0
*rTGA2.1*	2	0	1	0	1	0
*SaF*	1	0	1	0	1	0
*SaM*	1	0	0	0	1	0
*sd1*	1	0	0	0	0	0
*OsSSIIIa*	5	0	4	0	4	0
*Xa13*	2	0	1	0	1	1
*Xa1*	1	1	1	1	1	0
*Xa21*	0	0	0	0	0	0
*Xa26*	1	1	1	1	1	1
*Xa5*	1	0	2	0	2	0

## Discussion

In the present study, we conducted re-sequencing and genome-wide variation analysis of three famous representative restorer lines, namely IR24, MH63, and SH527, with the aim of uncover genetic variation at a genome-wide scale by using the Solexa sequencing technology. Identification of genome-wide SNPs, InDels, and SVs, as well as pattern analysis of restorer lines can provide valuable resources for future genetic studies and the molecular improvement of hybrid rice.

We firstly used the 9311 [5] and *Nipponbare* [4] sequence as the reference genome, respectively. The genome size of 9311 is 374,545,499, of which the effective size is 359,401,158 (excluding the N bases in the reference). On the other hand, the genome size of *Nipponbare* is 382,150,945, of which the effective size is 372,089,805. When the *Nipponbare* genome was used as the reference, the number of SNPs detected was noticeably higher (data not shown). However, quality of original sequence data such as mapped bases, sequencing depth, and coverage decreased, rendering the SNP data less reliable. Given that genetic variations between restorer lines, not the *japonica* and *indica* rice varieties, underlie the mechanism of their phenotypic differences, the 9311 genome sequence was then used as the only reference for detecting SNPs, InDels, and SVs, and for assembling the consensus sequence to exclude the large amount of background variations that account for differences between the *japonica* and *indica* rice varieties.

Interestingly, approximately 76,000, 71,000, and 76,000 heterozygous SNPs in IR24, MH63, and SH527, respectively, were identified throughout the whole rice genome, leading to an estimated heterozygosity rate of approximately 1.98–2.0×10^−4^, which is lower than that for other species, such as pandas [31] and humans [32]. The heterozygosity rate showed, to some extent, an un-purified genetic background of the sequenced rice varieties and indicated that the rice restorer lines still have high genetic variability, supporting the sporadic phenotypic variability of individuals observed within a rice line, even it is strictly self-pollinated. Thus we may speculate that, besides spontaneous mutations, genomic heterozygosity might also play a role in phenotypic variations. These results might also suggest that self-pollinated plants have the potential to maintain a relatively high heterozygosity rate. More plant lines should be studied to confirm this idea.

Here we report variations over the whole genome among elite rice restorer lines. Our results indicate that genetic variations among these lines, although far lower than those reported for a more diverse landrace population [10], are greater than expected, indicating a complicated genetic basis for the phenotypic diversity of the restorer lines. Although several candidate genes have been proposed to account for the varying performances of rice lines and selected for functional analysis, further analysis of more restorer lines is necessary to better understand the mechanism by which restorer lines are improved by breeding. Furthermore, several follow-up steps can be taken to pinpoint candidate genes that may contribute to phenotypic diversity in rice cultivars. This study therefore lays the groundwork for long-term efforts to uncover genes and alleles important for cultivar improvement in rice restorer lines.

## Materials and Methods

### Sampling

Seedlings of IR24, MH63, and SH527 and six other widely used CMS lines, namely, G3A, Zhongjiu A, II-32A, G46A, 92A, and Chuangu A, were planted in the experimental field of the Rice Research Institute, Sichuan Agricultural University, Wenjiang. When they reached the flowering stage, these three restorer lines were crossed with the six CMS lines to obtain the F_1_ hybrid rice. The three elite restorer lines, together with the F_1_ hybrid rice, were then planted in the following year for phenotypic evaluation and field test. All the restorer lines and the F_1_ hybrid rice were planted across 20 lines, with three replicates totaling 12 plants in each line. Eight middle plants of the 10 middle lines were surveyed, and data were recorded for statistical analysis. To compare the field performances of these elite restorer lines, we used a One-way ANOVA and LSD's test of DPS Software (http://www.chinadps.net/index.htm). To compare the contribution of restorer lines to their hybrids' field performances, we used a Two-way ANOVA and LSD's test of DPS Software (http://www.chinadps.net/index.htm) [33].

### DNA isolation and genome sequencing

Total genomic DNA was extracted from the leaf tissues of one individual for each line using a DNeasy Plant Mini Kit (Qiagen). The DNA of each line was then randomly fragmented. After electrophoresis, DNA fragments of the desired length were gel-purified. Adapter ligation and DNA cluster preparation were performed and subjected to Solexa sequencing.

### Read mapping

The raw pair-end (PE) sequencing reads were aligned to the 9311 reference genome sequence using SOAPaligner [13] under the following conditions: if an original read cannot be aligned to the reference sequence, the first nucleotide from the 5′ end and two nucleotides from the 3′ end will be deleted and then realigned to the reference. If the alignment still cannot be achieved, two more nucleotides from the 3′ end will be deleted. The procedure was repeated until the alignment was available or the read was less than 27 bp long. Average sequencing depth and coverage were calculated using the alignment results.

### Assembly of consensus sequences and SNP/InDel detection

Based on the alignment results, and taking into consideration the analysis of data characters, sequencing quality, and other factors influencing the experiments, a Bayesian model was applied to calculate the probability of genotypes with the actual data. The genotype with the highest probability was selected as the genotype of the sequencing individual at a specific locus, and a quality value was designated accordingly to reveal the accuracy of the genotype. Polymorphic loci against the reference sequence were selected from the consensus sequence and then filtered under certain requirements (e.g., the quality value must be greater than 20 and the result must be supported by at least two reads) using SOAPsnp [14]. Mapped reads that satisfied the PE requirements and contained alignment gaps at one end were also used to detect the short InDels. The maximum gap length allowed in the alignments was 5 bp. Gaps that were supported by at least three gapped PE reads were extracted in InDel calling.

### SV detection

According to the principle of PE sequencing, under normal situations, one read of PE should be aligned to the forward sequence and another should be aligned to the reverse. The distance between the two aligned positions at the reference should be in accordance with the insert size. Thus, the alignment of the two paired reads to the genome is regarded to be of normal direction and appropriate span. If the direction or span of the alignments of the two paired reads is different from that expected, then the region might have SVs. Abnormal PE alignments observed in our analysis were further analyzed by clustering and compared with previously defined SVs. In this manner, the SVs were detected using SOAPsv [14], with support from at least three abnormal PE reads. Currently, the types of SVs that can be detected include deletion, replication, reversion, and transposition, among others.

### SNP annotation

The localization of SNPs in coding regions, noncoding regions, start codons, stop codons, and splice sites were based on the annotation of gene models provided by the Rice Genome Sequencing Project of 9311 [34]. The characterization of synonymous or non-synonymous status of SNPs within the CDS was conducted using Genewise version 30 [35]. The GO/PFAM annotation data were further used to functionally annotate each gene [36].

### Variation frequency distribution

The frequencies of SNPs, InDels, and SVs for each sample were plotted over a 100 kb sliding window with a step size of 50 kb along each chromosome to explore the genomic distribution of DNA polymorphism in these lines [37]. The scanned regions were defined as high- or low-variation frequency regions if variation rates were higher than 4 fold or lower than 1/20th of the average rate over the whole genome (ARG), respectively. The deviation ratio (DR) of samples in a given window was first calculated as the sum of the ratio of each sample that deviated from the average rate, then the ARG was defined as the arithmetic average of all the windows across chromosomes.

### Variations between samples

The SNPs/InDels/SVs detected for each individual line were further compared between samples to identify the shared and unique SNP/InDel loci. Only those loci for which at least one effective sequence read was mapped for every individual were selected for comparison. A phylogenetic tree was constructed using the MEGA4 software [15] based on these data on SNPs.
